# Probiotic Properties and Optimization of Gamma-Aminobutyric Acid Production by *Lactiplantibacillus plantarum* FBT215

**DOI:** 10.4014/jmb.2204.04029

**Published:** 2022-05-13

**Authors:** Jaegon Kim, Myung-Hyun Lee, Min-Sun Kim, Gyeong-Hwuii Kim, Sung-Sik Yoon

**Affiliations:** Department of Biological Science and Technology, Yonsei University, Wonju 26493, Republic of Korea

**Keywords:** Gamma-aminobutyric acid, *Lactiplantibacillus plantarum*, probiotic properties, optimization, one-factor-at-a-time strategy, response surface methodology

## Abstract

Gamma-aminobutyric acid (GABA) improves various physiological illnesses, including diabetes, hypertension, depression, memory lapse, and insomnia in humans. Therefore, interest in the commercial production of GABA is steadily increasing. Lactic acid bacteria (LAB) have widely been reported as a GABA producer and are safe for human consumption. In this study, GABA-producing LAB were preliminarily identified and quantified via GABase assay. The acid and bile tolerance of the *L. plantarum* FBT215 strain were evaluated. The one-factor-at-a-time (OFAT) strategy was applied to determine the optimal conditions for GABA production using HPLC. Response surface methodology (RSM) with Box-Behnken design was used to predict the optimum GABA production. The strain FBT215 was shown to be acid and bile tolerant. The optimization of GABA production via the OFAT strategy resulted in an average GABA concentration of 1688.65 ± 14.29 μg/ml, while it was 1812.16 ± 23.16 μg/ml when RSM was applied. In conclusion, this study provides the optimum culture conditions for GABA production by the strain FBT215 and indicates that *L. plantarum* FBT215 is potentially promising for commercial functional probiotics with health claims.

## Introduction

Gamma-aminobutyric acid (GABA) is widespread in nature; it functions as an inhibitory neurotransmitter in the central nervous system [1]. GABA is present in plants, animals, and microorganisms, including bacteria and fungi [2]. Moreover, it influences various physiological responses such as antihypertensive effects, memory improvement, regulation of mood, and sleep induction [3]. Owing to these health benefits, GABA has gained wide attention [4].

GABA is commonly obtained through fermentation using mold, fungi, yeast, and bacteria [5]. Among these, lactic acid bacteria (LAB) have widely been studied as GABA producers not only because of their generally recognized as safe (GRAS) status but also their safety for human consumption the difficulty of human consumption of other microorganisms [6]. GABA-producing LAB are isolated from various fermented foods: *Levilactobacillus brevis* HY1, L-32, and 877G were obtained from kimchi [7-9]; *Lacticaseibacillus rhamnosus* 21D-B, *Lacticaseibacillus paracasei* 15C, and *Streptococcus thermophilus* 84C were isolated from cheese [10]; and *Lactobacillus senmaizukei* sp. nov. was obtained from pickle [11]. Optimizing microbial GABA production is indispensable for manufacturing commercial GABA products [7, 12, 13].

Microbial GABA production is influenced by factors, such as fermentation time, initial pH, glutamate concentration, and medium composition [14]. These factors could be optimized through a one-factor-at-a-time (OFAT) strategy or response surface methodology (RSM) [7, 13]. In the conventional OFAT strategy, only one independent factor is varied while keeping the others constant. Optimization of GABA production by LAB using the conventional OFAT approach is insufficient; it does not consider interactions among the individual factors in a complex system [15-18]. RSM, a statistical method, is a design of experiments (DoE) commonly used to evaluate the effects of different factors [19]. It can be used to predict an optimal condition through a sequence of designed experiments [20]. Therefore, RSM saves experimental resources by reducing the number of experiments for optimization [21].

In this study, GABA-producing *Lactiplantibacillus plantarum* (formerly *Lactobacillus plantarum*) FBT215 was isolated from kimchi. The basic probiotic properties of *L. plantarum* FBT215 were investigated. The optimal fermentation conditions, including temperature, initial pH, carbon source, nitrogen source, and supplementation of L-monosodium glutamate (MSG) and pyridoxal-5’-phosphate (PLP), were determined using the OFAT strategy. A statistical method based on the Box-Behnken design (BBD) was used to determine the optimal concentration of each factor. The optimal conditions for GABA production by *L. plantarum* FBT215 were investigated through these two methods and compared.

## Materials and Methods

### Isolation of GABA-Producing Lactic acid Bacteria

Ten varieties of Korean fermented foods were collected from the traditional market in Gangwon-do, Republic of Korea. Each sample was suspended in 0.85% (w/v) NaCl (saline) and spun down to remove the food particles. The supernatant was filtered through a 5 μm filter paper (Toyo Roshi Kaisha, Ltd., Japan). The GABA-producing LAB were screened as described previously [22]. Briefly, the samples were centrifuged at 3,000 ×*g* for 10 min. The bacterial pellet was resuspended in de Man, Rogosa, and Sharpe broth (MRS broth; Becton, Dickinson and Company, USA) containing 50 mM MSG (pH 6.5; Sigma-Aldrich, USA) and incubated at 37°C for 3 h. The pH of the culture broth was adjusted to 4.0, followed by incubation at 37°C for 3 h. After incubation, each 10-fold diluted solution was inoculated on MRS agar plates containing bromocresol purple (BCP) and 0.02% (w/v) sodium azide (NaN_3_; Sigma-Aldrich); the plates were cultured at 37°C for 48 ± 3 h. Yellowish colonies grown on the plates were selected as the putative GABA-producing LAB strain.

### GABase Assay

Putative GABA-producing LAB isolates were quantified through a spectrophotometric assay using the GABase enzyme. The isolated sample was centrifuged at 3,000 ×*g* for 10 min. The culture supernatants were treated with Carrez solution I (0.25 M potassium ferrocyanide; Sigma-Aldrich) and II (1.0 M zinc acetate; Samchun Chemical Co., Republic of Korea, 2% (v/v) glacial acetic acid; Daejung Chemical Co., Republic of Korea) for 30 min. The samples were then centrifuged at 10,000 ×*g* for 1 min. The reaction mixture for quantification consisted of 2.3 ml of 100 mM potassium pyrophosphate (Daejung Chemical Co.) buffer, 0.1 ml of 100 mM 2-mercaptoethanol solution (Sigma-Aldrich), 0.15 ml of 25 mM β-nicotinamide adenine dinucleotide phosphate (β-NADP; Sigma-Aldrich), 100 mM α-ketoglutarate (Sigma-Aldrich), and 0.3 ml of 10-fold diluted sample. 2 units/ml of GABase enzyme (Sigma-Aldrich) was added to each sample, and incubated at 25°C for 1 h. GABA concentration was determined based on the absorbance at 340 nm using a spectrophotometer (Multiskan Sky; Thermo Fisher Scientific, USA).

### 16S rDNA Sequencing and Identification of Adhesion-Related Gene Sequence

The universal primer set 27F (5’-AGAGTTTGATCMTGGCTCAG-3’) and 1492R (5’-GGTTACCTTGTT ACGACTT-3’) were used to identify the GABA producers [23]. SrtAF (5’-ATGAAGTCCAAGCAACA-3’) and SrtAR (5’- TTAATATTTGTTATTAAAATGACTTG-3’) were used for the amplification of the sortase A (SrtA). The PCR cycling conditions were: initial denaturation at 95°C/5 min, 35 cycles of denaturation at 95°C/30 sec, annealing at 55°C/30 sec, and extension at 72°C/30 sec, and a final extension at 72°C/7 min. DNA sequencing was performed at Macrogen (Republic of Korea). Sequence comparisons were performed using the Basic Local Alignment Search Tool (BLAST) available at the National Center for Biotechnology Information (NCBI; National Institutes of Health, USA). Sequence alignments were performed using BioEdit (Ibis Biosciences, USA).

### Acid and Bile Tolerance

*L. plantarum* FBT215 was cultured in MRS broth at 37°C for 24 h. The culture broth was centrifuged at 6,000 ×*g* for 5 min, and the bacterial pellet was resuspended in saline. The acid tolerance was determined after adjusting the pH of the suspensions to pH 2.5, 3.0, and 6.0 with 1% (w/v) pepsin (Junsei Chemical Co., Japan). The viable cell count was measured every 1 h for 2 h on MRS agar. The bacterial culture was incubated in pH-adjusted MRS broth (pH 3.0) at 37°C for 2 h to determine bile tolerance. Subsequently, the culture broth was centrifuged at 6,000 ×*g* for 5 min; the medium was replaced with an equal volume of MRS broth with 0.3% (w/v) bile (oxgall, Sigma-Aldrich). The viable cell count was measured every 3 h for 6 h on MRS agar.

### HPLC

GABA production was analyzed using HPLC (1260 Infinity series; Agilent Technologies) and a Poroshell120 HPH-C18 (4.6 mm × 150 mm × 4 μm; Agilent technologies). The pre-column *ortho*-phthalaldehyde derivatization method was used for analyzing GABA production. The automated injection program was performed as described by Agilent Technologies (USA), with slight modifications. In brief, solvent A contained 10 mM Na_2_HPO_4_, 10 mM Na_2_B_4_O_7_, and 5 mM NaN_3_; solvent B contained acetonitrile: methanol: water (45:45:10, v:v:v). The solutions were filtered through a 0.20 μm membrane (Hyundai Micro Co., LTD, Republic of Korea) using vacuum filtration. Sonication (JAC-1505; Kodo, Hwaseong-si, Republic of Korea) was performed for the bubble decay process. The isocratic elution conditions were: 2% solvent B from 0 min to 0.35 min, 57% solvent B from 0.35 min to 13.4 min, 100% solvent B from 13.4 min to 20.3 min, and 0% mobile phase B from 20.3 min to 23.0 min.

### Optimization of GABA Production via OFAT Strategy

The OFAT strategy was used to determine the optimal conditions for GABA production by *L. plantarum* FBT215; the optimal temperature, initial pH, and fermentation time in MRS broth were determined. The effect of culture temperature in modified-MRS broth containing 50 mM MSG was evaluated at 25°C, 30°C, 37°C, 40°C, and 45°C. The influence of initial pH on GABA production was investigated at pH 3.5, 4.5, 5.5, 6.5, 7.5, 8.5, and 9.5. The optimal fermentation time was measured every 3 h for 12 h and then every 12 h until 96 h. The 2% glucose in MRS broth (pH 7.5) was replaced with different carbon sources (2% w/v; fructose, sucrose, lactose, galactose, xylose, maltose, mannitol, and lactulose). The 1% proteose-peptone No. 3 and 1% beef extract in MRS broth (pH 7.5) were replaced with different nitrogen sources (2% w/v; peptone, tryptone, soytone, proteose-peptone No.3, malt extract, and beef extract). The 2% glucose in MRS broth (pH 7.5) was replaced with three fructose-containing polymers (2% w/v; fructooligosaccharides, inulin, and raffinose) to evaluate the availability of prebiotics as a carbon source. The MSG concentrations (25–250 mM) and PLP concentrations (0–50 mM) were individually optimized.

### Optimization of GABA Production via RSM

The RSM package in R software, version 4.1.2 (The R Foundation, Austria), was used to predict the optimal conditions for GABA production. RSM using the BBD model was performed, as described previously [7], with slight modifications. In brief, carbon concentration, nitrogen concentration, and initial pH were chosen as major factors influencing GABA production. Each factor was transformed into 3 coded levels (-1, 0, and 1). A second-order model was used to fit the data for the Y responses (GABA concentration). The Quadratic equation is as follows:

$$Y=\beta_{0}+\sum {}_{i=1}^{3}\beta_{i}X_{i}+\sum {}_{i=1}^{2}\sum {}_{j=i+1}^{3}\beta_{ij}X_{i}X_{j}+\sum {}_{i=1}^{3}\beta_{ii}X_{i}^{2}+e$$

*β*_0_ is constant; *β*_i_, *β*_j_, and *β*_ii_ are coefficients of variables. *X*_i_ and *X*_j_ indicate the levels of independent variables. Analysis of variance and regression analysis were performed using R.

### Sequence Accession Number

The 16S rRNA gene sequence of *L. plantarum* FBT215 (accession no. OL587487) is available on GenBank (NCBI).

### Statistical Analysis

Assays were performed in triplicate, and the results were analyzed using IBM SPSS Statistics 25 (IBM Corp., USA). Data are presented as mean ± standard deviation in bar charts. One-way ANOVA and Tukey’s multiple range tests were used to evaluate the significant differences (*p* < 0.01) among the groups.

## Results

### Isolation and Identification of GABA-Producing LAB

**Fig. 1:**
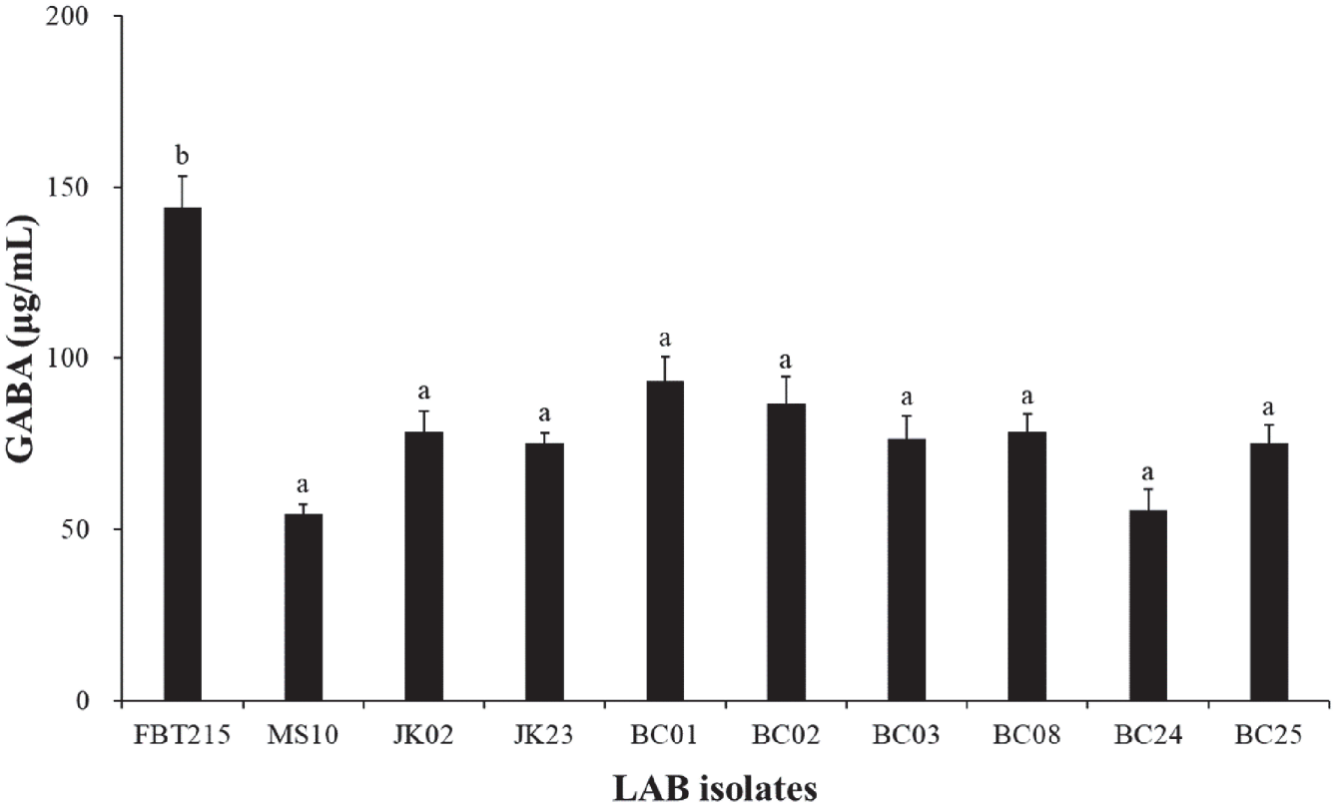
The amount of GABA produced by LAB isolates cultured in modified-MRS broth supplemented with 50 mM MSG at 37°C for 48 h. The GABA concentration was quantified using the GABase assay. GABA, gammaaminobutyric acid; LAB, lactic acid bacteria; MSG, monosodium glutamate.

Potential GABA-producing LAB strains were isolated from 10 varieties of Korean fermented foods. GABA production from the isolates was evaluated using a GABase assay. Ten isolates produced high amounts of GABA (54.37 ± 5.44 μg/ml to 144.02 ± 14.40 μg/ml, Fig. 1); among them, isolate FBT215, which is the highest GABA producer at 144.02 ± 14.40 μg/ml, was selected for further experiments. 16S rRNA gene sequencing identified isolate FBT215 as *L. plantarum*.

### Probiotic Properties: Acid Tolerance, Bile Tolerance, and Identification of an Adhesion-Related Gene

**Fig. 2:**
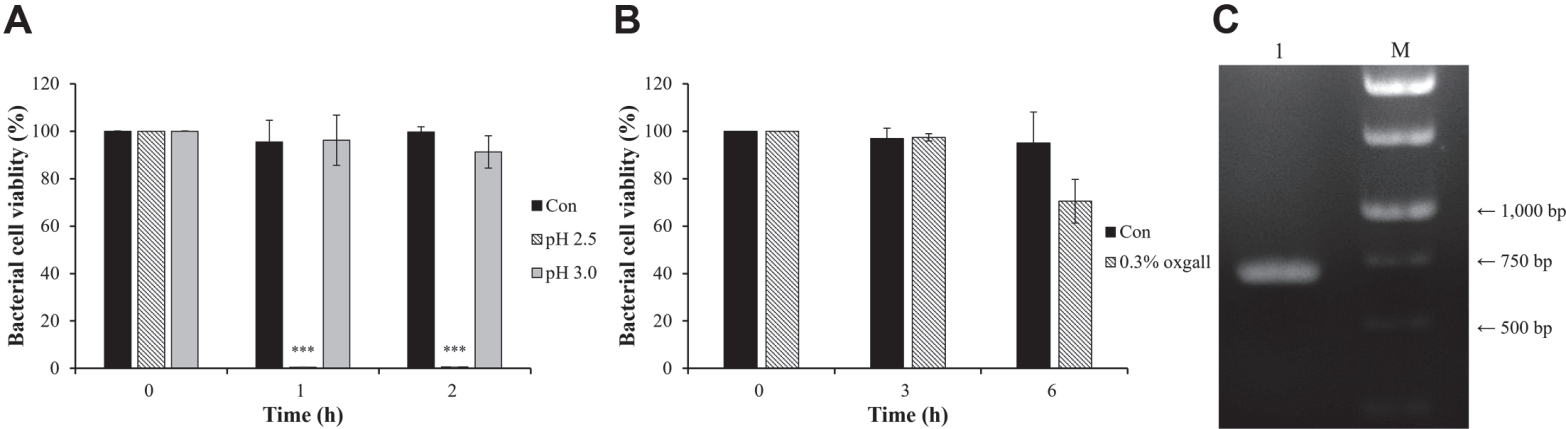
Probiotic properties of *Lactiplantibacillus plantarum* FBT215. (**A**) Acid tolerance of *L. plantarum* FBT215 at pH 2.5, 3.0, and 6.0 (control). The cell viability was evaluated every hour for 2 h on an MRS agar plate. (**B**) Bile tolerance of *L. plantarum* FBT215. The cell viability was calculated every 3 h for 6 h on an MRS agar plate. (**C**) Gel electrophoresis of sortase A coding gene. The amplicon was visualized using 1.2% (w/v) agarose gel. Lane M, molecular mass marker (Thermo Fisher Scientific, SM0311); 1, sortase A amplicon.

At pH 2.5, the viability of *L. plantarum* FBT215 decreased significantly to 0.56 ± 0.11% and 0.61 ± 0.10%, after 1 and 2 h of incubation (*p* < 0.001, Fig. 2A), respectively. However, at pH 3.0, the viability was 96.1 ± 10.6% and 91.3± 9.20%, respectively. *L. plantarum* FBT215 survived in 0.3% (w/v) bile for 6 h (Fig. 2B). The viability was not significantly different compared to that of the control group. The putative adhesion-related enzyme, sortase A, was amplified via PCR; an approximately 700 bp amplicon was obtained (Fig. 2C).

### Optimization of GABA Production via OFAT Strategy

**Fig. 3:**
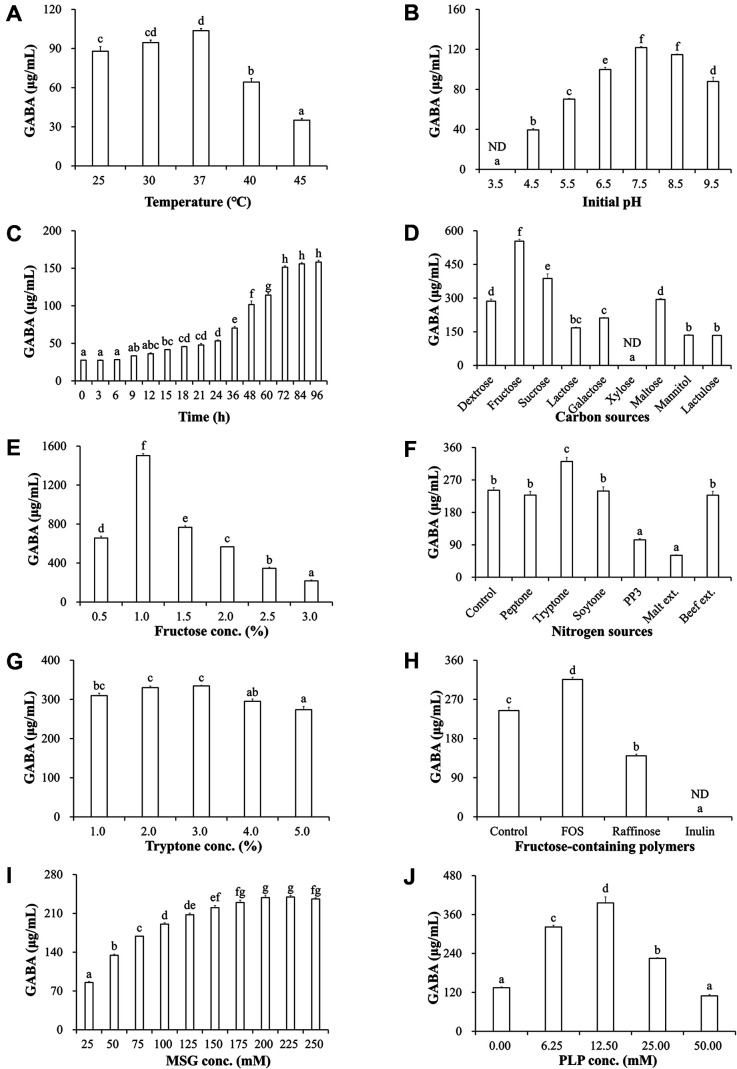
The concentration of GABA produced by *Lactiplantibacillus plantarum* FBT215 in modified-MRS broth. (**A**) The optimal temperature was determined by evaluating the GABA production at 25°C, 30°C, 37°C, 40°C, and 45°C in MRS broth supplemented with 50 mM MSG. (**B**) The optimal pH was determined by investigating the GABA production, at different pH conditions (pH 3.5-9.5), at 37°C in MRS broth supplemented with 50 mM MSG (**C**). The optimal incubation time was investigated for 96 h in MRS broth supplemented with 50 mM MSG at 37°C. (**D**) The optimum carbon source among nine different carbon sources (glucose, fructose, sucrose, lactose, galactose, xylose, mannose, mannitol, and lactulose) was determined by culturing in MRS broth supplemented with 50 mM MSG, at 37°C and pH 7.5, for 72 h. (**E**) The optimal fructose concentration in the range of 1−5% was investigated by culturing in MRS broth supplemented with 50 mM MSG, at 37°C and pH 7.5, for 72 h. (**F**) The optimum nitrogen source among seven different nitrogen sources (control, peptone, tryptone, soytone, proteose-peptone No. 3, malt extract, and beef extract) was determined by culturing in MRS broth supplemented with 50 mM MSG, at 37°C and pH 7.5, for 72 h. (**G**) The optimal tryptone concentration in the range of 1−5% was determined by culturing in MRS broth supplemented with 50 mM MSG, at 37°C and pH 7.5, for 72 h. (**H**) The optimal fructose-containing polymers among three different sources (Fructooligosaccharides, raffinose, and inulin) were determined by culturing in MRS broth supplemented with 50 mM MSG, at 37°C and pH 7.5, for 72 h. (**I**) The effect of MSG supplementation was evaluated in the range of 25-250 mM by culturing in MRS broth at 37°C and pH 7.5 for 72 h. (**J**) The effect of PLP supplementation was evaluated in the range of 0–50 mM by culturing in MRS broth supplemented with 50 mM MSG, at 37°C and pH 7.5, for 72 h. MSG, monosodium glutamate; PLP, pyridoxal 5’-phosphate.

GABA production was analyzed using the OFAT strategy and HPLC to determine the optimal culture conditions for *L. plantarum* FBT215. The optimal culture temperature for GABA production was 37°C, at which the GABA concentration was 103.67 ± 1.65 μg/ml (Fig. 3A). The GABA content significantly decreased at temperatures greater than or less than 37°C. The optimal pH for GABA production was measured in the pH range of 4.5–9.5. The optimal initial pH was 7.5 and 8.5 (121.76 ± 1.14 μg/ml and 114.75 ± 0.56 μg/ml, respectively, Fig. 3B); and was not detected at pH 3.5. The GABA production increased steadily from 9 h (33.28 ± 0.43 μg/ml) to 72 h (151. 42 ± 1.96 μg/ml); however, it remained stable after 72 h (Fig. 3C).

The environmental factors were maintained at the optimal levels for the subsequent experiments. Several nutritional factors were assessed to enhance GABA production. Fructose was the best carbon source (553.57 ± 8.65 μg/ml); however, GABA production was enhanced in sucrose-containing broth (387.36 ± 19.85 μg/ml) when compared to that with glucose (286.17 ± 9.00 μg/ml), which represents the commercial MRS broth (Fig. 3D). However, lactose, galactose, xylose, maltose, mannitol, and lactulose did not improve GABA production compared to media with glucose. The optimal fructose concentration was 1% (w/v, 1502.40 ± 21.04 μg/ml, Fig. 3E). Tryptone was the best nitrogen source for GABA production (321.21 ± 11.53 μg/ml, Fig. 3F); peptone, soytone, proteose-peptone No. 3, malt extract, and beef extract did not improve GABA production compared to that in the commercial MRS broth (241.53 ± 7.19 μg/ml). The optimal tryptone concentration was 2% (w/v) and 3% (w/v) (330.17 ± 3.83 μg/ml and 334.41 ± 1.63 μg/ml, respectively, Fig. 3G). GABA production decreased with the addition of inulin and raffinose; however, the addition of fructooligosaccharides (FOS) enhanced GABA production (Fig. 3H).

The influence of MSG and PLP was evaluated in MRS-based broth. GABA production increased depending on MSG concentration (25 mM to 200 mM; 85.26 ± 1.88 μg/ml to 238.43 ± 4.44 μg/ml, respectively, Fig. 3I). The GABA production was maintained at 225 mM MSG; however, it was slightly decreased at 250 mM (236.15 ± 2.12 μg/ml). The addition of PLP influenced the GABA production by *L. plantarum* FBT215. The optimal PLP concentration was 12.50 mM (396.00 ± 18.26 μg/ml, Fig. 3J).

### Optimization of GABA Production via RSM

The optimal nutritional factors for GABA production were determined using the OFAT strategy; fructose and tryptone were the best carbon and nitrogen sources, respectively. Subsequently, the initial pH and the two nutritional factors were optimized using the RSM with the BBD model. The three variables were coded to three levels, and GABA production was calculated based on the average of triplicates. Data were analyzed using the quadratic regression model, and the critical variables were identified through backward elimination. As a result, the following equation was used.

$$Y=2213.93+69.37X_{1}-47.91X_{2}-292.85X_{3}-93.74X_{1}X_{2}+42.24X_{1}X_{3}+37.68X_{2}X_{3}-949.28X_{1}^{2}-960.19X_{2}^{2}-921.66X_{3}^{2}$$

**Fig. 4:**
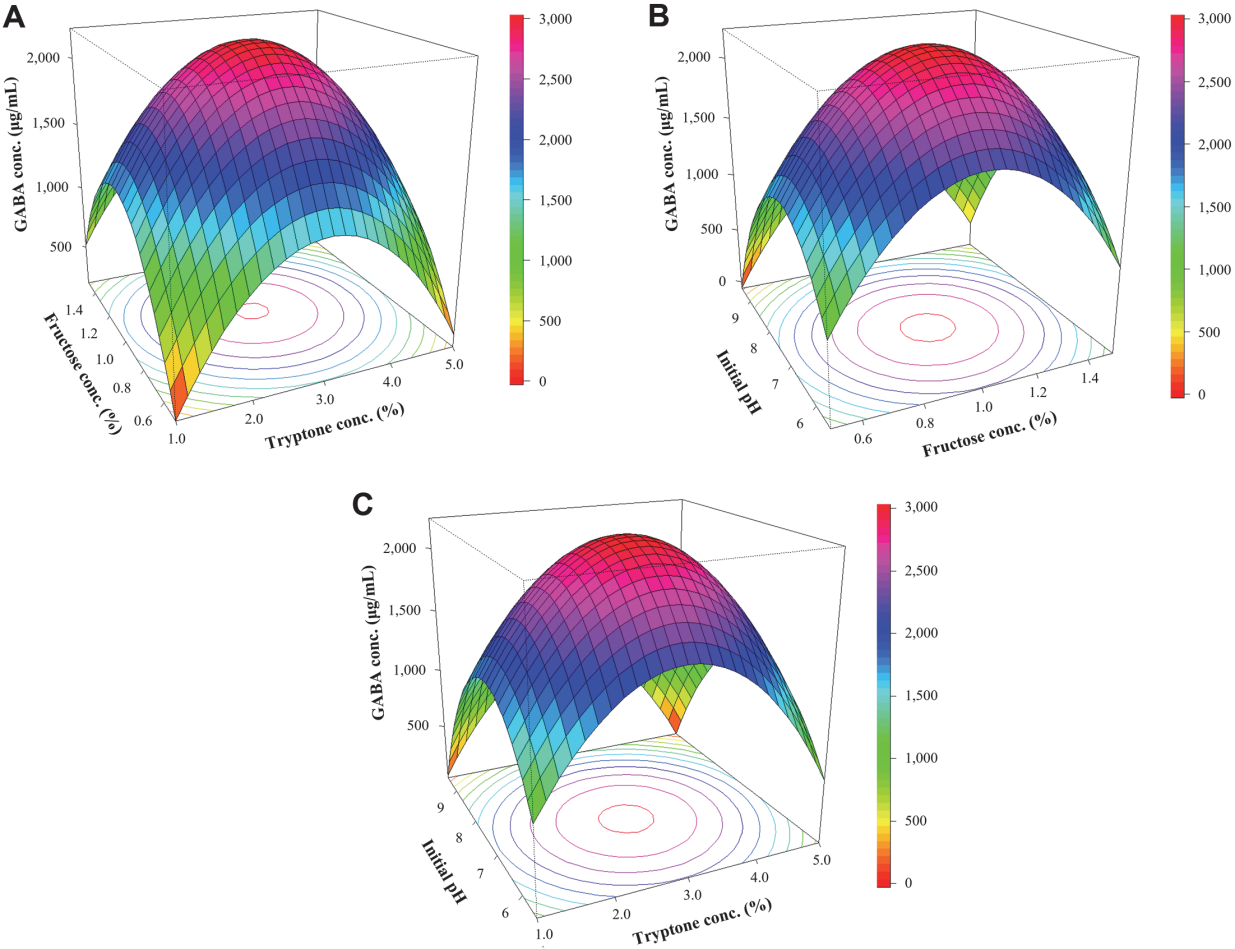
Three-dimensional response surface plots illustrating the effect of each variable on GABA production by *L. plantarum* FBT215. (**A**) GABA concentration by the interaction between fructose concentration and tryptone concentration. (**B**) GABA concentration by the interaction between fructose concentration and initial pH. (**C**) GABA concentration by the interaction between tryptone concentration and initial pH.

*X*_1_, *X*_2_, and *X*_3_ indicate the concentration of fructose, tryptone, and the initial pH, respectively. This model’s multiple R-squared and adjusted R-squared value was 0.9903 and 0.9730, respectively. The value of Pr (>F) of lack of fit was 0.057 (Table 2), suggesting that this regression model was suitable for analyzing the GABA production. The responses considering the interaction of two variables with the other variable fixed at the optimal point were illustrated using a three-dimensional response surface graph. The effect of fructose and tryptone concentrations on GABA production at an initial pH of 7.18 was analyzed (Fig. 4A). The relationship between fructose concentrations and initial pH was analyzed at a tryptone concentration of 2.94% (w/v, Fig. 4B). Additionally, the effect of tryptone concentration and initial pH was analyzed at a fructose concentration of 1.02% (w/v, Fig. 4C). The optimum conditions for maximum GABA production were determined using the quadratic regression model equation and response surface graph; they were 1.02% fructose, 2.94% tryptone, and an initial pH of 7.18. The GABA content was 2239.07 μg/ml at the optimum point.

## Discussion

GABA is a major inhibitory neurotransmitter in the CNS; its beneficial effects are widely recognized. In this study, we investigated the basic probiotic properties of GABA-producing *L. plantarum* FBT215. In addition, the optimal conditions for GABA production by *L. plantarum* FBT215 were determined using the OFAT strategy and the RSM method; the optimal conditions determined through the two methods were compared.

The survival of probiotic bacteria in the gastrointestinal tract is incumbent on their acid and bile tolerance [24]. *L. rhamnosus* GG, a probiotic bacterium, was unable to grow at pH < 3.0 and could tolerate 0.15% (w/v) bile salts [25]. Similarly, *L. plantarum* FBT215 was vulnerable at low pH (< 3.0) but had tolerance to bile salts (Fig. 2). The pH in the human stomach ranges from 1.0 to 2.0; however, it could be 3.0 or higher in the presence of food [26]. Therefore, the application of *L. plantarum* FBT215 as probiotics could be more efficient when coated with an acid-tolerance agent or consumed immediately after a meal. *srtA* codes sortase A; it recognizes LPxTG sorting motifs and influences the adhesion of *L. acidophilus* ATCC 4356. [27]. SrtA deletion significantly decreases the surface exposure of mannose-specific adhesins; this could explain the effects on mannose-specific adhesion [28]. The complete sequence of *L. plantarum* FBT215 SrtA was identified and was consistent with that of *SrtA* from *L. plantarum* ATCC 202195 (Accession No. QVG76613.1) and *L. plantarum* ATCC 14917 (Accession No. EFK27984.1). This work has opened new avenues to study the association between SrtA and the cell adhesion capacity of *L. plantarum* FBT215; the LPxTG sorting motifs and mannose-specific adhesin-related genes can be explored further.

The optimal conditions for efficient GABA production by *L. plantarum* FBT215 under various environmental and nutritional conditions were determined using HPLC. *L. plantarum* is a mesophilic bacterium; the optimal growth temperature is 37°C in MRS broth [29]. Microbial GABA production is efficient at a low pH; however, GABA production is optimal at neutral pH in some *Lactobacillus* spp. [30]. The severe growth retardation of LAB at low pH could inadequate GABA production. The GABA production in *L. plantarum* FBT215 was decreased under acidic conditions and was maximum at pH 7.5 and 8.5 (Fig. 3B). The preferred carbon source for GABA production varies with the LAB strain: 1% (w/v) glucose plus 1% fructose for *L. brevis* CRL 2013 [31], 3% glucose for *L. plantarum* N5 [32], 2% maltose for *L. brevis* HYE1 [33], and 2% glucose for *L. plantarum* KCTC 3103 [13]. *L. plantarum* FBT215 produced the highest amounts of GABA with 1% fructose (Fig. 3E). Using this strain would be industrially advantageous due to the lower carbon source consumption than other LAB. The optimal nitrogen source for GABA production by *L. plantarum* FBT215 was 2% tryptone (Fig. 3G). These results contradict the earlier finding that yeast extract was the optimal nitrogen source [16, 17, 32]. However, yeast produces various biologically active ingredients containing GABA and glutamate [34, 35]. Therefore, yeast extract was considered a growth factor for the precise analysis of GABA production by *L. plantarum* FBT215. Consequently, the optimum carbon and nitrogen sources for GABA production by *L. plantarum* FBT215 were 1% fructose and 2% tryptone, respectively. Fructose-based carbon sources, except inulin, were metabolized for GABA synthesis in *L. plantarum* FBT215. FOS and raffinose lyase could be encoded in the putative probiotics; further studies are warranted. The addition of PLP, a co-factor of glutamate decarboxylase (GAD), increased GABA production (Fig. 3J) and is a major factor influencing GABA production [36, 37].

The OFAT strategy is traditionally employed to optimize the responses of interest. Several studies aimed to optimize the conditions for enhancing GABA production by LAB [15-18]. However, the method has limitations because the interactions of individual factors cannot be considered in a complex system; it can only be evaluated at specific points [33]. Therefore, RSM with the CCD model was assessed for optimizing GABA production in an *L. fermentum* isolate. Three variables were analyzed, *i.e.*, glucose concentration, MSG concentration, and incubation time [38]. GABA production by *Lactobacillus* sp. *Makhdzir Naser*-1 was optimized with a focus on temperature, pH, glutamic acid concentration, and incubation time [39]. In this study, optimization of GABA production by *L. plantarum* FBT215 was performed using RSM with a BBD model. The actual GABA content at the optimal conditions predicted using the conventional and statistical methods was significantly different (*p* < 0.05), at 1688.65 ± 14.29 μg/ml and 1812.16 ± 23.16 μg/ml, respectively (data not shown). The actual GABA content via RSM with the BBD model was 80.93% of the predicted value. Therefore, RSM with the BBD model is more reliable for optimizing the GABA production in *L. plantarum* FBT215.

In the beginning, GABA was chemically synthesized to meet demands, but this synthesis method was replaced due to the higher yields and lower costs of the biosynthetic process [40]. In this study, optimization of GABA production by *L. plantarum* FBT215 was investigated under various conditions. LAB have widely been studied as GABA producers not only because of their GRAS status but also the difficulty of human consumption of other microorganisms. Although optimization of GABA-producing LAB has already been reported in previous studies, it is vital to screen new resources because of the differences in the fermentation profiles of individual LAB [41]. Among LAB, *L. plantarum* has been investigated as a key species for GABA production [42]. *L. plantarum* FBT215 produced a high amount of GABA in MRS-based medium compared to that by others: 0.74 g/l for *L. plantarum* Taj-Apis362 [43], 1.5 g/l for *L. plantarum* K154 [44], 0.1 g/l for *L. plantarum* NMZ [45], and 0.6 g/l for *L. plantarum* NTU 102 [46]. GABA has long been thought to be unable to cross the blood-brain barrier; however, it is suggested that GABA exerts veridical effects on the brain possibly via the enteric nervous system [4]. Studies in animals have reported that the gut microbiota can regulate GABAergic neurotransmission through the vagus nerve, which is the main pathway from the abdominal cavity to the brain [47, 48]. Currently, the number of patients affected by physiological disorders is gradually increasing, and it has been suggested that GABA could potentially support a role for dysregulated GABAergic functioning in neural circuits [49]. Therefore, further studies are needed on GABA production by *L. plantarum* FBT215, which could exert a therapeutic effect on physiological illnesses in mammalian models.

In summary, the basic probiotic properties of the LAB strain *L. plantarum* FBT215 were investigated. GABA production was optimized using the OFAT strategy and RSM with a BBD model. This study can provide culture conditions for commercial functional probiotics with health claims in vitro. Further studies are warranted to optimize the various culture conditions to enhance GABA production and understand the in vivo physiological effects of GABA produced by *L. plantarum* FBT215.

## Figures and Tables

**Table 1:** Box-Behnken design matrix and the predicted GABA concentrations.

Factors	Symbol	Coded variable levels		

		-1	0	1

Fructose conc. (%, w/v)	*X*_1_	0.5	1	1.5
Tryptone conc. (%, w/v)	*X*_2_	1	3	5
Initial pH	*X*_3_	5.5	7.5	9.5


Run	*X*_1_	*X*_2_	*X*_3_	*Y* (μg/ml)

1	0	-1	1	40.13
2	0	1	-1	548.68
3	0	-1	-1	690.12
4	0	1	1	49.41
5	-1	0	1	36.38
6	1	0	-1	565.14
7	-1	0	-1	717.65
8	1	0	1	52.84
9	-1	1	0	162.66
10	1	-1	0	633.74
11	-1	-1	0	100.74
12	1	1	0	320.72
13	0	0	0	2221.16
14	0	0	0	2168.85
15	0	0	0	2251.79

**Table 2:** ANOVA for GABA production.

Factors	Sum of square	Mean square	F value	Pr(>F)
FO(*X*_1_, *X*_2_, *X*_3_)	742972	247657	13.601	0.0077
TWI(*X*_1_, *X*_2_, *X*_3_)	47962	15987	0.878	0.5117
PQ(*X*_1_, *X*_2_, *X*_3_)	8552763	2850921	156.564	2.31E-05
Residuals	91046	18209		
Lack of fit	87529	29176	16.588	0.0573
Pure error	3518	1759		
Multiple R^2^:	0.9903		Adjusted R^2^:	0.9730
